# Non-Rainfall Moisture Activates Fungal Decomposition of Surface Litter in the Namib Sand Sea

**DOI:** 10.1371/journal.pone.0126977

**Published:** 2015-05-15

**Authors:** Kathryn Jacobson, Anne van Diepeningen, Sarah Evans, Rachel Fritts, Philipp Gemmel, Chris Marsho, Mary Seely, Anthony Wenndt, Xiaoxuan Yang, Peter Jacobson

**Affiliations:** 1 Biology Department, Grinnell College, Grinnell, Iowa, United States of America; 2 CBS-KNAW Fungal Biodiversity Centre, Utrecht, The Netherlands; 3 Kellogg Biological Station, Michigan State University, Hickory Corners, Michigan, United States of America; 4 Gobabeb Research and Training Centre, Gobabeb, Namibia; North Carolina State University, UNITED STATES

## Abstract

The hyper-arid western Namib Sand Sea (mean annual rainfall 0–17 mm) is a detritus-based ecosystem in which primary production is driven by large, but infrequent rainfall events. A diverse Namib detritivore community is sustained by minimal moisture inputs from rain and fog. The decomposition of plant material in the Namib Sand Sea (NSS) has long been assumed to be the province of these detritivores, with beetles and termites alone accounting for the majority of litter losses. We have found that a mesophilic Ascomycete community, which responds within minutes to moisture availability, is present on litter of the perennial Namib dune grass *Stipagrostis sabulicola*. Important fungal traits that allow survival and decomposition in this hyper-arid environment with intense desiccation, temperature and UV radiation stress are darkly-pigmented hyphae, a thermal range that includes the relatively low temperature experienced during fog and dew, and an ability to survive daily thermal and desiccation stress at temperatures as high as 50°C for five hours. While rainfall is very limited in this area, fog and high humidity provide regular periods (≥ 1 hour) of sufficient moisture that can wet substrates and hence allow fungal growth on average every 3 days. Furthermore, these fungi reduce the C/N ratio of the litter by a factor of two and thus detritivores, like the termite *Psammotermes allocerus*, favor fungal-infected litter parts. Our studies show that despite the hyper-aridity of the NSS, fungi are a key component of energy flow and biogeochemical cycling that should be accounted for in models addressing how the NSS ecosystem will respond to projected climate changes which may alter precipitation, dew and fog regimes.

## Introduction

Across all ecosystems, the proportion of primary production that is consumed as litter, rather than actively-transpiring vegetation is estimated to be as high as 50–99% [1]. In arid ecosystems, where primary production enters the system in infrequent pulse-driven episodes associated with rain events, the proportion of primary productivity moving through the detrital community is particularly high [2,3]. Litter that becomes incorporated into the soil is decomposed by bacteria and fungi [4], and is also consumed by diverse communities of detritivores which are then eaten by higher trophic levels of invertebrate and vertebrates carnivores. Polis (1991) [2] estimates that in arid ecosystems, only 12–33% of energy flows via plant-herbivore-carnivore and that the majority goes via plant litter-detritivore-carnivore pathways.

**Fig 1:**
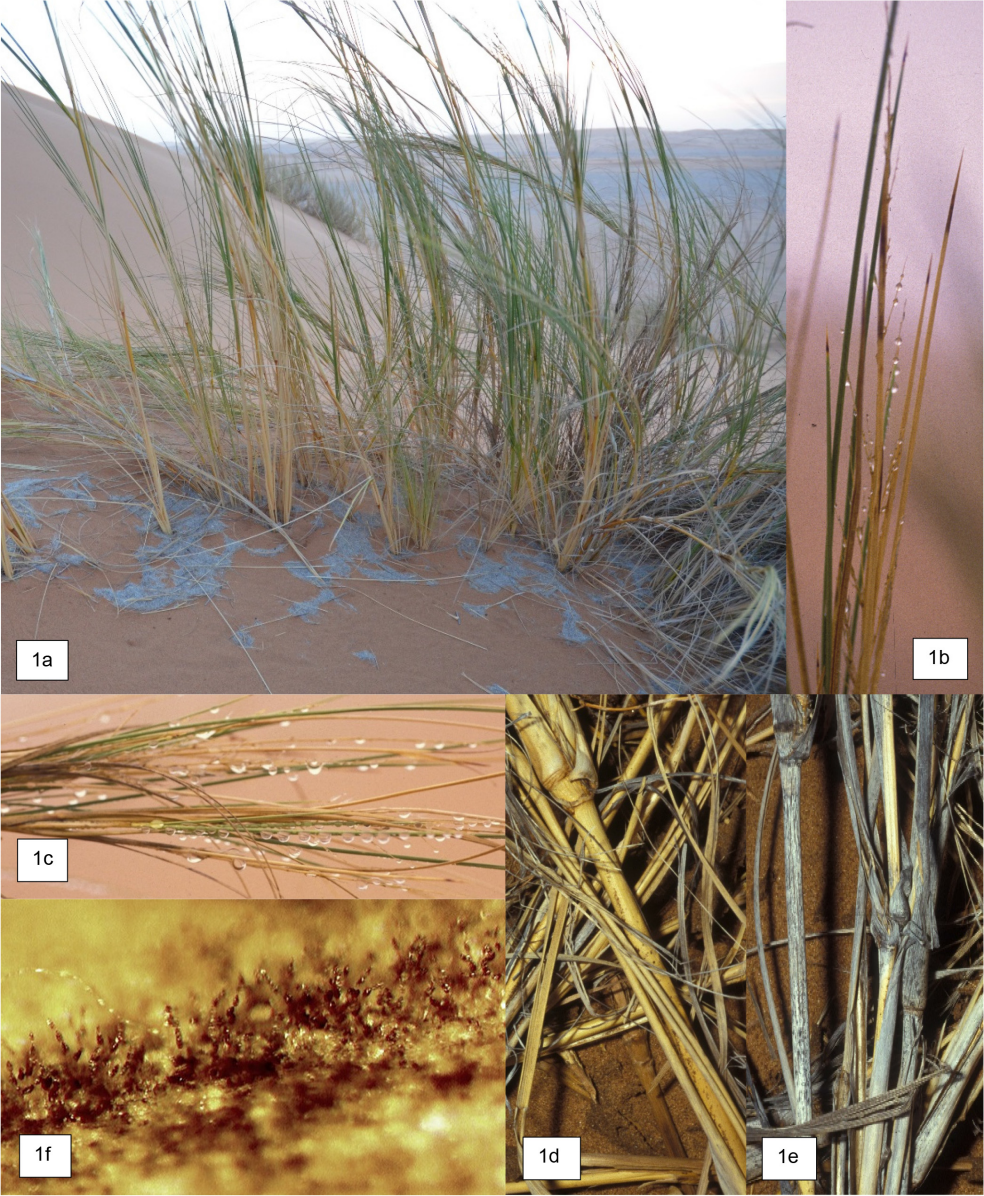
Surface litter of the fog-adapted grass, *S*. *sabulicola*, is colonized by fungi. Living perennial plant with golden-yellow senescent stems (standing litter) and wind-blown detritus (fine grey surface litter) (1a); fog moisture precipitates on the entire length of the grooved upright stems and may be funnelled to the roots (1b); moisture also collects on leaning grass stems and inflorescences and drips to the sand surface where it can be consumed by beetles and reptiles (1c). Even in the absence of rain events, standing senescent stems and inflorescence litter changes color from golden-yellow (1d), to deep grey (1e). Wetting the grey litter, intended to simulate precipitating fog, resulted in visible fungal growth and spore production within 6 hours (1f).

The hyper-arid Namib Sand Sea (NSS) is a classic detritus-driven ecosystem. A year after a significant rain pulse, 92–94% of the resulting vegetation biomass is wind-blown detritus that sustains detritivores over a multi-year period [5,6]. These detritivores (e.g. termites, tenebrionid beetles, microarthropods and ants) feed a diverse array of higher trophic levels of arachnids, reptiles, small mammals and birds [5,7]. During the intervening dry years (mean annual rainfall <20 mm), a large proportion of the moisture needs of the NSS fauna are met with fog originating from the Atlantic Ocean. The unique fog-harvesting adaptations of beetles and lizards are particularly well-described [8]. Primary production of the dominant perennial dune grass, *S*. *sabulicola* (Fig 1A), is also sustained by fog. While plants only germinate in response to rains greater than 20–25 mm [9], continuous plant growth and seed production is sustained by fog, with plants known to persist for over 35 years in large hummocks [9]. Stems of *S*. *sabulicola* are grooved, and moisture precipitating from fog collects on the tall (1.5–2.0 m) upright stems (Fig 1B) and is funnelled down to the roots [10]. Partially buried senescent stems that are well-anchored remain upright for long periods of time and continue to trap fog moisture. Water also collects on leaning grass stems and inflorescences (Fig 1C) and drips to the sand surface where it may be consumed by beetles and reptiles.

Even in the absence of fog formation, high humidity can be a biologically-significant moisture source in deserts. In the NSS, the moisture content of dry grass can reach 27% at a relative humidity of 90% [11]. During every month of the year, the prevailing wind direction is from the southwest, bringing moist air from the Atlantic Ocean that can raise humidity to high levels during the early morning hours [12]. In a single year Henschel and Seely [8] documented high humidity (>95%) on 189 days, averaging 5.3 hours per episode. Perennial senescent grasses absorb this moisture and are subsequently consumed by herbivores, supplementing their moisture balance [12].

The hyper-arid NSS surface environment, with daily thermal fluxes on the sand surface ranging from 15–55°C [6,10], is a highly stressful environment which necessitates adaptive responses by the biota. All of the grasses in the NSS use C4 metabolism and most of the fauna are fossorial and/or nocturnal, with only a few species actively foraging during the heat of the day [13]. While fog, dew and high humidity may sustain a diverse fauna and flora, the extremely low rainfall of the NSS was thought to preclude microbes [14] and thus nutrient turnover was long assumed to be a dry-season process, driven primarily by detritivores [3,13]. Gut microbes of detritivores were assumed to be primary agents of mineralization [15], with decomposition of wind-blown detritus and buried litter by free-living microbes described by Seely and Pallett [13] as “extremely erratic.” Termites accounted for 65% of the buried detritus loss in one study during a dry period [7], supporting the hypothesis that the NSS lacked a free-living, microbially-mediated decomposition loop [16]. However, 82–100% of buried cellulose substrates were rapidly mineralized by microbes and detritivores following rains greater than 9 mm [4], illustrating that decomposition of buried litter in the NSS is both a dry- and “wet”-season process, as it is in other arid ecosystems [17], and that free-living microbes, including large desert-adapted macrofungi (Basidiomycota) [18], are likely important drivers of rapid below–ground decomposition, as in other deserts [19]. As in many other hyper-arid regions, free-living prokaryotic microbes have now also been described from the soils and hypolithic environments of the Namib Desert [20].

In contrast to the below-ground environment, it has been assumed that the surface environment in the NSS (and other drylands) is too extreme to support microbial decomposition. However, we observed in the field that standing senescent *S*. *sabulicola* stem and inflorescence litter change color from golden-yellow (Fig 1D) to deep grey (Fig 1E), within a year of plant senescence, even in the absence of rain events. Microscopic examination revealed that this color change of the grass stem surface coincides with the increased presence of darkly-pigmented fungal hyphae and spores. Gentle spritzing of the litter with water in the lab, intended to simulate precipitating fog, resulted in visible growth and spore production after just six hours (Fig 1F). Furthermore, Dirks et al. [21] observed litter mass losses in the absence of precipitation events in the Mediterranean and proposed that in addition to loss via UV degradation, that the decomposer microbial community was causing mass loss in response to events of elevated humidity (>90% humidity). We thus hypothesized [H_1_] that frequent small precipitation events (fog, dew and rainfall) activate fungi on standing litter, which contribute to decomposition processes while the material is still above-ground. To address this hypothesis we first quantified the frequency of precipitation (rain or precipitating fog) and high humidity (>95%) events that could result in fungal activity in the Namib. We then conducted moistening and drying trials of incubated grey *S*. *sabulicola* litter samples and measured CO_2_ fluxes to quantify the response time and activity of the microbial community. Thereafter we applied a fungicide to the litter to estimate the proportion of measured microbial respiration that was fungal. In addition, we isolated pure cultures of the fungal taxa colonizing the standing litter in order to identify the fungi involved, to determine their cardinal temperatures (thermal optima and lower and upper thermal range limits), and to conduct experiments to determine whether they could survive the daily thermal fluctuations observed in the NSS.

**Fig 2:**
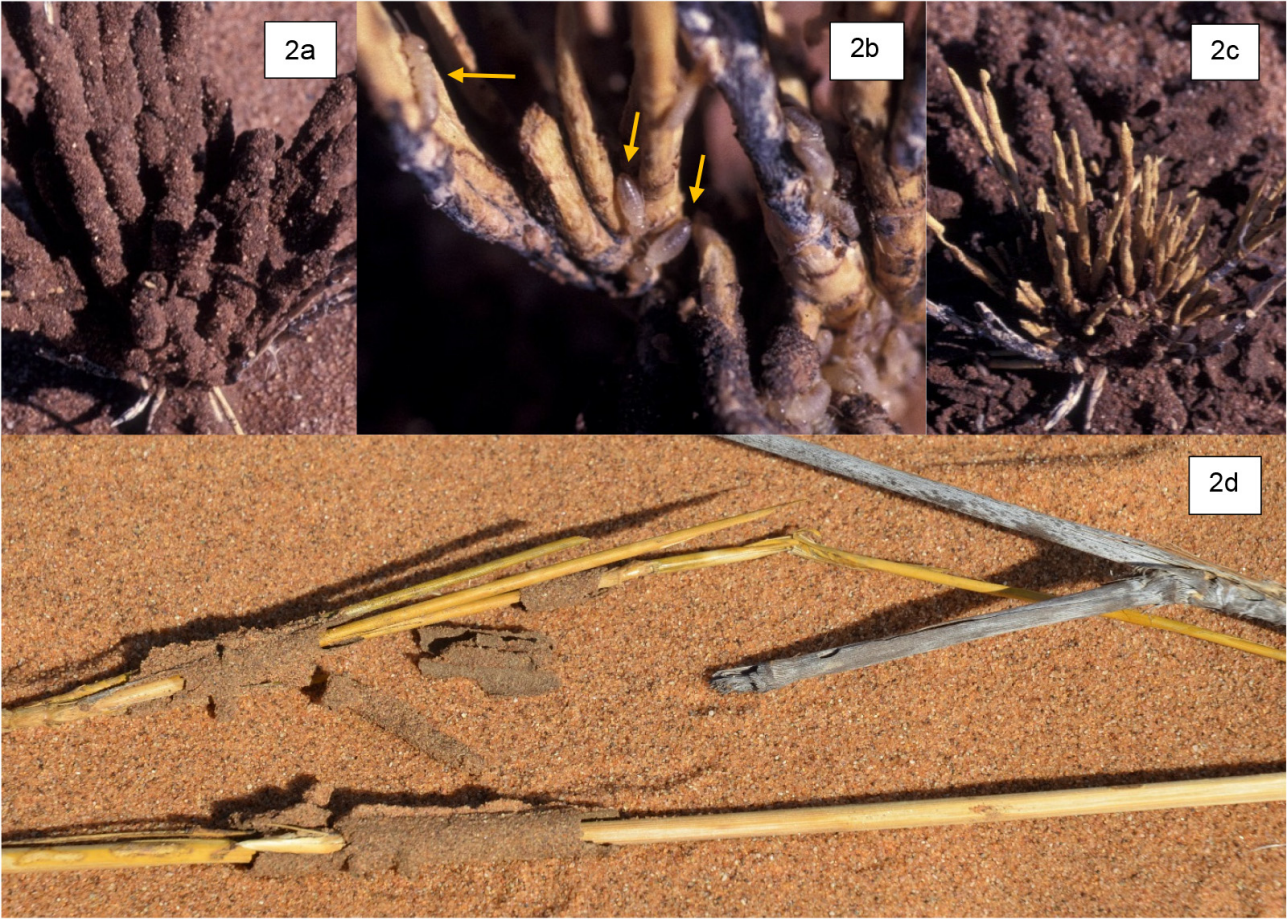
Termites (*Psammotermes allocerus*) consume fungal-colonized litter. Perennial *Stipagrostis ciliata* litter with termite casts intact (2a). Termite cast has been removed, showing termites (arrows) stripping the grey outer layer, leaving the golden-yellow interior (2b). Note the small remaining portion of grey litter in the upper center. Standing litter from 2a with cast removed showing that all grey outer surface has been stripped by termites inside the cast (2c). Examples of *S*. *sabulicola* stem litter used in the C/N analysis (2d): grey litter (upper right) was compared with golden-yellow stripped material (left and bottom) from inside termite casts and golden-yellow standing litter as seen in Fig 1A.

Furthermore, we hypothesized [H_2_] that fog-activated colonization and decomposition of aboveground litter has implications for other decomposition processes, mediated through altered stoichiometry. This is based on our observation that the termite, *Psammotermes allocerus*, selectively grazes the outer grey layer of the stems of perennial *Stipagrostis* species (Fig 2A–2D). Carbon:nitrogen (C/N) ratios of standing senescent grasses in deserts are high: 71.5–115.4 [22], and as in other deserts, soil nitrogen levels in the NSS are very low [3,5,23,24]. We thus predicted that fungal colonization of this litter enriches the N content making it a desirable nutritional resource for detritivores. Accordingly, we determined the C/N ratio of the external grey surface material (Figs 1E and 2D) that the termites were consuming to assess whether it differed from that of the remaining uneaten yellow cores found in the casts (Fig 2B and 2D), and from yellow grass stems not yet visibly colonized by fungi (Fig 1D).

## Methods

### 1. Analysis of moisture inputs in the Namib Sand Sea

We examined a 7-year period (1982–89) of continuous daily recordings of humidity fluxes and precipitation to determine the frequency of high humidity, precipitating fog and rain events. The moisture data was recorded on autographic drum recorders at the Gobabeb Research and Training Centre (GRTC) [25]. Rainfall was recorded in a metal rain gauge [26]; fog was monitored using a cylindrical metal screen (10 cm diameter, 22 cm tall) attached to a separate rain gauge [25], and humidity was measured with a dry-wet bulb, each instrument attached to a separate autographic data recorder. We retrieved the associated data scrolls from the Gobabeb Library Archive and analysed a 2556 day period for the daily sources and amounts of fog, rainfall and high humidity, the number of days with six or more hours of >95% humidity, and the number of days with more than one hour of >95% humidity.

### 2. Respiratory responses of microbial community to rewetting and subsequent drying

The research permit for field work within the Namib-Naukluft Park was obtained from the Namibian Ministry of Environment and Tourism (Permit # 1960/2014). We cut standing, dry senescent grey and golden-yellow stem litter from living *S*. *sabulicola* grass hummocks in the late afternoon of a hot dry day on Station Dune ~1 km south of the GRTC (S 23.588148° E 15.052270°), and stored them in sterile Whirlpak bags at room temperature. We conducted lab-based respiration experiments on pieces of this stem litter, measuring CO_2_ flux on a mass basis using an infrared gas analyser (PP Systems, Model EGM-2) fitted with a 10-ml syringe within a closed, desiccated recirculation loop.

For the wetting experiment, the stem litter (mean = 4.6 mm diameter) was cut into pieces 4–5 cm long, dried at 40°C in a drying oven for two hours, and weighed on an analytical balance to the nearest tenth of a milligram. Ten replicates were placed in separate sterile syringes that could be connected to the instrument. The dry litter samples were moistened by spraying each replicate with sterile deionized water from a misting bottle until they were visibly moistened (standardized to five sprays in rapid succession) to simulate a precipitating fog or dew deposition, and thereafter returned to the syringes. CO_2_ flux measurements were started 5 minutes after moistening and repeated at 0.5, 1, 3 and 10 hours. The flux rate was determined by plotting the rate of change in the CO_2_ concentration of the chamber at 8 s intervals over a 64 s period. Flux rates were expressed on a dry weight basis as μg CO_2_-C g^-1^ litter min^-1^ [27].

Five pieces of the litter used in the wetting experiment were examined and photographed with Leica dissecting and compound microscopes (1,000x) to qualitatively assess surficial microbial growth after the 10-hour wetting experiment.

To determine whether high humidity could result in microbial activity, we placed the same five pieces of stem litter in a well-sealed desiccator with the base filled with deionized water, to achieve a saturated atmosphere [28]. We also placed thinner leaves (≈0.2–0.3 mm thick, dried and weighed) in the desiccator to determine how rapidly they could imbibe moisture and become active. Over the course of 2 hours, we periodically measured moisture uptake by the substrate as a function of mass gained and determined CO_2_ flux as above.

To examine how drying affected respiration rates, the five remaining moistened replicates were weighed, and then dried in their respective syringes in a drying oven at 40°C over a 12-hour period until all moisture was eliminated. Measurements of respiration rate and mass were recorded every 2 hours. Moisture content (g H_2_0 g^-1^ litter) was calculated relative to the initial dry weight of the litter sample prior to wetting.

### 3. Respiratory responses of microbial community to application of fungicide

Ten 4–5 cm replicates of the dry senescent grey stem litter, sampled and stored as in the wetting experiment, were dried at 40°C for 2 hours, weighed, moistened with sterile deionized water and placed in separate sterile syringes as above. The CO_2_ flux measurements were started 5 minutes after moistening and repeated at 0.5, 1, 3 and 10 hour as in the initial wetting experiment. Thereafter all ten replicate samples were dried at 40°C for 6 hours and weighed to ensure dryness. Five of the dried replicates were randomly chosen to serve as the Captan control, and were again moistened with sterile deionized water. The remaining five were moistened with a solution of Captan (3.9 g L^-1^ prepared in sterile deionized water). Flux measurements of the five samples from each treatment were started at 5 minutes, and repeated, calculated and reported as above. Comparisons of mean flux rates among the treatment groups were made using one-way analysis of variance (ANOVA) followed by post-hoc Tukey’s tests using Minitab 16 (Minitab Inc., State College, PA).

### 4. Taxonomy and traits of fungal taxa colonizing *S*. *sabulicola*

The fungi were isolated from dried standing *S*. *sabulicola* (n = 3) at two sites subject to frequent fog: Station Dune near the GRTC (S 23.588148° E 15.052270°) and Nara Valley (S 23.515254° E 14.959081°). The sampling was not intended to rigorously assess richness and diversity, but rather to provide a first approximation of the taxa involved. The grass material was cut from standing grey stems and immediately placed in sterile Whirl-Pak bags which were subsequently maintained at ambient temperature. Small pieces (5–10 mm) were placed on malt or water agar and incubated at 30°C until hyphal extension into the agar was sufficient for sub-culturing. Isolates were made to individual malt agar plates and stored permanently at -80°C in the Grinnell College Fungal Culture Collection until further use. DNA was extracted from liquid malt cultures by the CTAB-based method [29]. The quality of genomic DNA was tested by running 2–3 μL on a 0.8% agarose gel, while DNA was quantified with a NanoDrop 2000 spectrophotometer (Thermo Fisher, Wilmington, U.S.A.). DNA samples were stored at −20°C until use.

The barcoding regions ITS and 18S ribosomal RNA (SSU) were used for fungal identification: for the ITS region primers ITS1 and ITS4 were used, while for SSU the primer pair NS5/NS6 was used [30]. For some species, these sequences didn’t resolve to the species level and additional gene fragments were amplified: for *Aspergillus* species we used part of the calmodulin gene amplified with primers cmd5 and cmd6 [31], and for *Fusarium* species the EF1 and EF2 primers [32] were used. PCR amplifications were done as described for the respective primers in 12.5 μl volumes. Amplicons were purified with Sephadex G-50 fine (HE Healthcare Bio-Sciences AB, Uppsala, Sweden).

Sequencing was done directly on the purified PCR product using the ABI prism BigDye terminator cycle sequence kit (Applied Biosystems, Foster City, USA) and analyzed on an ABI Prism 3730XL Sequencer. Sequences were edited using SeqMan in the Lasergene software (DNASTAR, Madison, WI, USA). The sequences were identified by BLASTing to Genbank sequences (http://www.ncbi.nlm.nih.gov/) and the CBS database (http://www.cbs.knaw.nl/). The *Fusarium* species were resolved using the Fusarium MLST database (http://www.cbs.knaw.nl/fusarium/ [32]). Positive identification at the species level was defined as identity scores >98%. The sequences were deposited in GenBank (KP941081–KP941108).

Cardinal temperatures (thermal optima and high and low ranges) of the isolates were based on three replicates at temperatures ranging from 5–55°C, which represents the extreme thermal limits experienced on the sand surface [6]. The thermal optimum was the temperature at which an isolate achieved the greatest radial growth measured from a central inoculation point after seven days. Growth rate at the optimal temperature was calculated over a five-day period, as the total radial growth that occurred between the second and seventh days. The thermal range was the minimum and maximum temperatures at which an isolate showed hyphal growth into the agar at seven days. Student’s *t*-tests were used to compare growth rates among groups of species using Minitab 16 (Minitab Inc., State College, PA).

To determine whether the isolates could survive the extreme daily thermal flux in the Namib Sand Sea, we programmed a Percival growth chamber to mimic a diel sand-surface thermal cycle [6]: 15°C for 2 hours, 1-hour increments of increasing temperature from 20–45°C, 50°C for 5 hours, 1-hour increments of decreasing temperature from 45–25°C, and 20°C for 6 hours. Incandescent lights were off during the 8 hours of 15–20°C temperatures that corresponded to night-time temperatures experienced 16–17 December. Relative humidity in the chamber was stable at 30% during the night time temperatures and decreased to ~5% during the hot day time hours. Replicate isolates (n = 3) on malt agar were subjected to this fluctuating thermal regime for seven days after an initial 24 hours of growth at 30°C, with measurements of radial growth and growth rate as above.

### 5. C/N analysis of *S*. *sabulicola* litter consumed by *Psammotermes allocerus*

Because we observed termites eating the grey outer layer of *S*. *sabulicola* stem litter and leaving the inner yellow core intact, we determined the C/N ratio of this grey material relative to that of the unconsumed inner core. To insure that the outer cortex of the stem did not have an inherently different C/N ratio relative to the inner core, we also analysed yellow litter that had not yet discoloured and did not exhibit surficial fungal colonization. At five different grass hummocks on Station Dune at Gobabeb we thus obtained three sample types: yellow, stripped and unconsumed stem litter core from within a termite cast; yellow stem litter and grey stem litter. Samples of each of the three litter types were shaved from the parent material with a razor blade. The outer grey layer that was shaved off extended 1–1.5 mm into the stem cortex and we therefore shaved off a similar depth of material from the yellow stem litter and the unconsumed yellow stem core. All samples were ground to a fine powder by initial treatment of shavings in a Wiley mill followed by grinding using a Retsch ball mill. Approximately 5 mg of the dried sample was analysed for C and N content using a Thermo Finnigan EA 1112 CN elemental analyser. Comparisons of mean C/N ratios among litter sample types were made using one-way analysis of variance (ANOVA) followed by post-hoc Tukey’s tests using Minitab 16 (Minitab Inc., State College, PA.).

## Results

### 1. Frequency and duration of moisture inputs at Gobabeb

Over the 2556 day period of our analysis (1982–1989), mean annual rainfall was 14 mm and rain was recorded on 51/2556 days (2%) (Table 1). Less than 12 mm fell on 98% of those days, and most rainfall events were very small (0.1–2.0 mm on 68% of the 51 days). Of the total annual precipitation recorded during our 7-year period of analysis, 84% was derived from fog in events averaging 1.4 mm on 88% of the days during which precipitation occurred. The vast majority of precipitation events, both rain and fog, were extremely small (means: 1.4 mm/fog event, 2.2 mm/rain event) but occurred, on average, every 6–7 days. High humidity, which lasted for at least six hours (including that associated with rain and precipitating fog), occurred on average 76 days/year or about once every 4–5 days. Finally, high humidity sustained for at least one hour occurred on average every 3 days.

**Table 1:** Surface moisture availability at Gobabeb Research and Training Centre, Namib Desert.

	Mean	Median	Range
**Annual rainfall (mm)**	14.1	15.0	4.9–23.5
**Annual fog (mm)**	69.2	64.3	55.9–98.3
**Total annual precipitation (mm)**	83.3	77.5	64.2–112.8
**Rainfall/event (mm)**	2.2	1.7	1.0–4.7
**Precipitating fog/event (mm)**	1.4	1.4	0.9–1.8
**Annual rainfall days**	7	7	4–13
**Annual precipitating fog days**	52	50	38–70
**Total annual precipitation days**	59	57	45–83
**Days/year with >6 hours of >95% humidity**	76	72	40–138
**Days/year with >1 hour of >95% humidity**	129	121	73–187

High humidity (>95%), rainfall and fog precipitation over a seven-year continuous period (1982–1989) at GRTC. Data retrieved from the long-term data records of the GRTC First Order Meteorological Station, obtained on autographic drum recorders.

## 2. Wetting and drying trials

**Fig 3:**
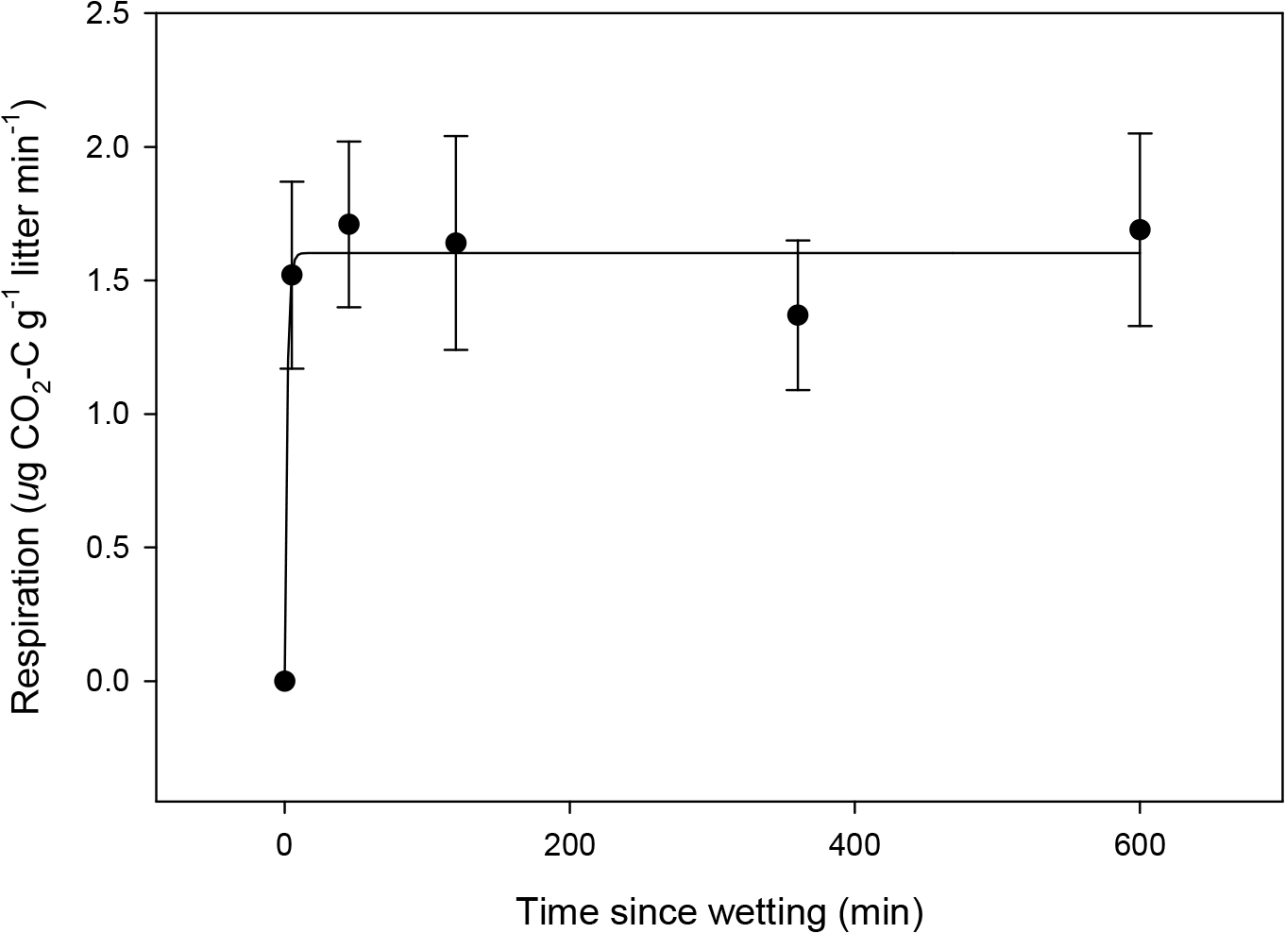
Rapid respiratory response to wetting of colonized litter. Respiration response of moistened grey *S*. *sabulicola* standing litter over ten hours. An initial reading of the dried material was taken prior to time 0, when the material was visibly moistened by spraying (n = 10, error bars = 1 SE)**.**

**Fig 4:**
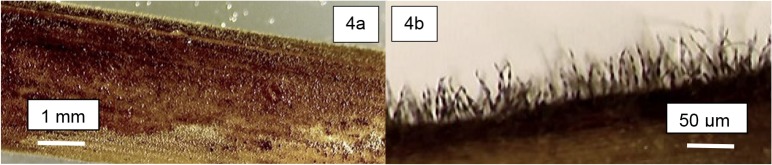
Fungal growth and sporulation on *S*. *sabulicola* litter. Fungal hyphae growing on *S*. *sabulicola* substrates after 10 hours incubation (4a: 20x magnification, 4b: 400x magnification).

**Fig 5:**
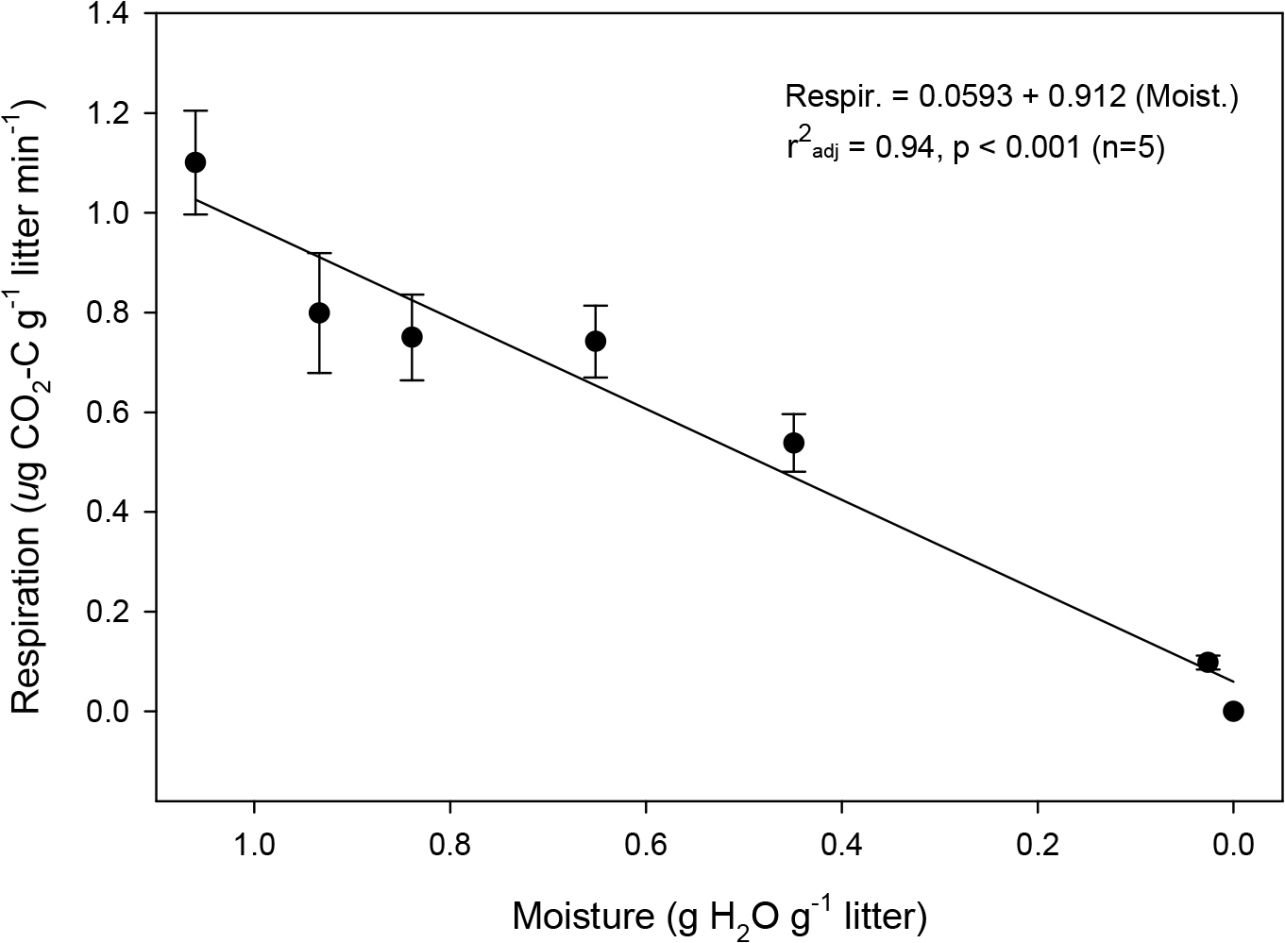
Litter respiration declines with drying. Respiration fluxes fall linearly as litter dries (n = 10, error bars = 1 SE)**.**

CO_2_ flux was detected within 5 minutes of moistening *S*. *sabulicola* litter. Mean flux rate changed from 0 in the dry material to 1.5 μg CO_2_-C g litter^-1^ min^-1^ and was sustained near this level for the 10-hour duration of the experiment (Fig 3). At the conclusion of this period, the litter was fuzzy (Fig 4A) and fungal growth and sporulation were observed (Fig 4B). Microscopic examination of the litter surface revealed abundant fungal hyphae and spores, but no evidence of Actinomycete hyphae or other prokaryotic cells. When the litter replicates were dried at 40°C after the wetting experiment, respiration rate showed a significant linear decline in response to declining moisture content (Fig 5), with the respiration rate still approximately 50% of the maximum when the litter had lost half the absorbed moisture.

In the high humidity experiment, the water content of the litter stems increased very slowly, reaching only 10.5% after 2 hours, with no detectable CO_2_ flux. In contrast, the thinner leaves (seen in Fig 1C–1E), reached a moisture content of 30.3% and exhibited a flux rate of 0.99 μg CO_2_-C g litter^-1^ min^-1^ after 2 hours.

### 3. Respiratory response of the microbial community to application of fungicide

**Fig 6:**
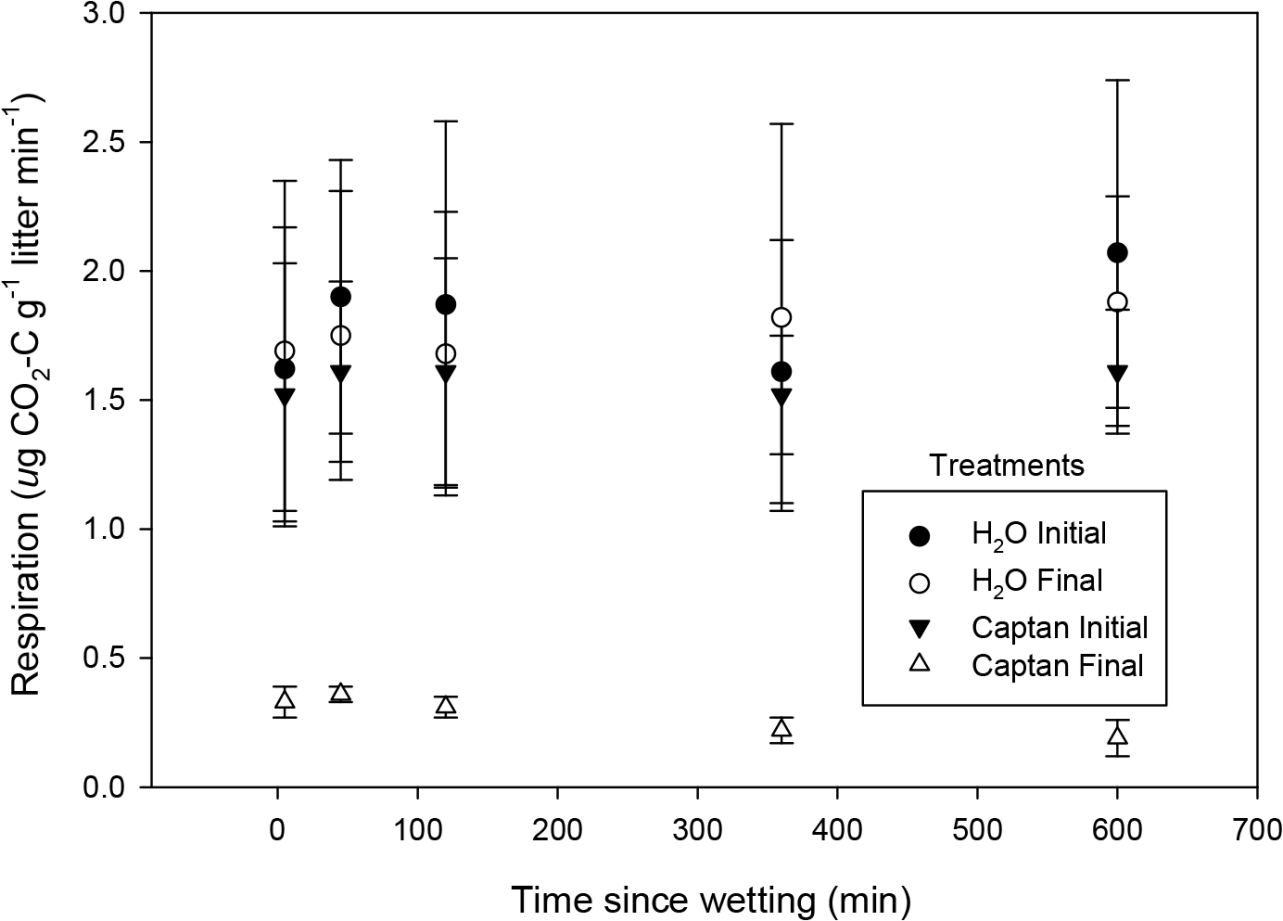
Fungicide suppresses litter respiratory response to wetting. Respiration response of litter moistened twice with water, with drying after initial wetting (“H_2_0 initial” and “H_2_O final”); compared to response of litter moistened initially with water (“Captan Initial”) followed by wetting with Captan solution (“Captan Final”). The responses of the first three treatments are not significantly different from one another, but are significantly different from the Captan treatment (p<0.001). (n = 5, error bars = 1 SE).

As in the wetting experiment, all ten samples became metabolically active (based on sustained CO_2_ flux) within five minutes of moistening with water, and maintained this activity for the duration of the ten-hour control experiment (Fig 6). In the subsequent experiment, performed with these same litter samples, there was no significant difference in response of the “Captan Initial” samples that were again moistened with water, relative to the initial ten samples. In contrast, CO_2_ flux in the five samples that were previously exposed and then re-wet with the Captan solution (“Captan Final”) rose rapidly to only approximately 20% of the control’s mean flux rate, suggesting that the majority (approximately 80%) of the CO_2_ flux measured in the wetting experiments could be attributed to microbes that were targeted by the Captan. In addition to supressing fungal respiration, Captan can also affect bacteria, including Actinomycetes, among the most desiccation-resistant prokaryotes. Thus, 80% may be an overestimation of the fungal contribution to respiration, although microscopic examination of the litter revealed no prokaryotic cells. The remaining proportion (approximately 20%) is likely attributable to microbes not susceptible to this fungicide.

### 4. Characteristics of the fungal taxa isolated from *S*. *sabulicola* standing litter

All species identified are known soil or plant litter saprophytes, except for *Fusarium thapsinum*, which is known only from living plants as an endophyte or plant pathogen (Table 2). Half of the isolates are known plant endophytes: the three *Fusarium* species, *Aspergillus niger*, *A*. *terreus* and *Thielavia subthermophila*. In addition, the three *Fusarium* species are also known to be plant pathogens of various monocot crop species. All species, except recently described *T*. *intermedia*, are known from arid or semi-arid habitats, but only three species are exclusively known from deserts (*Chaetomium atrobrunneum*, *C*. *strumarium*, *T*. *microspora)*.

**Table 2:** *Stipagrostis sabulicola* litter fungal taxa and traits.

Fungal taxa	KJCC Strain no. Genbank: ITS Accession no. SSU Accession no.	OGT (°C)	Thermal range (°C)	Growth rate at OGT (mm d^-1^)	% OGT reduction with daily thermal cycling	Nutrient acquisition strategy	Known habitat
**Eurotiales, Trichocomaceae**							
*Aspergillus nidulans* (Eidam) G. Winter	KJ111 KP941094 KP941108	35	15–50	4.2	60	**Saprophyte:** soil, plant litter [33]	**Desert** [34–42]; **Cosmopolitan** [33,43]
*Aspergillus niger* Tiegh.	KJ108 KP941093 KP941107	30–35	15–50	2.1	62	**Saprophyte:** soil [33,44], plant litter [45,46]; **Endophyte** [47]	**Desert** [34,35,37,38, 41,42,47–54]**; Cosmopolitan** [33,42,43,55]
*Aspergillus terreus* Thom.	KJ121 KP941092 KP941106	30–35	15–40	1.8	43	S**aprophyte**: soil [33], plant litter [45]; **Endophyte** [47]	**Deser**t [35,39–41,47,51–3,56,57]; **Cosmopolitan** [33,43,55]
**Dothiales, Dothioraceae**							
*Aureobasidium pullulans* (de Bary) G. Arnaud	KJ106 KP941091 KP941105	25	5–35	1.8	88	**Saprophyte**: plant litter [58,59]; **Endophyte** [58,60,61]	**Desert** [34,37,38,41,50,52,60–62]**; Cosmopolitan** [58,63]
**Sordariales, Chaetomiaceae**							
*Chaetomium atrobrunneum* L.M. Ames	KJ112 KP941081 KP941095	30–35	10–45	12.1	38	**Saprophyte:** soil [64]	**Desert** [64]
*Chaetomium brasiliense* Bat. & Pontual	KJ130 KP941090 KP941104	30–35	10–40	3.6	33	**Saprophyte**: soil [36,38,55]	**Desert** [36,38]; **Tropical** [55]
*Chaetomium strumarium* (J. N. Rai, J. P. Tewari & Mukerji) P. F. Cannon	KJ125 KP941089 KP941103	40	10–50	12.1	49	**Saprophyte**: biocrust [65]	**Deser**t [65]
*Thielavia arenaria* Mouch.	KJ119 KP941088 KP941102	40	10–45	4.8	60	**Saprophyte**: soil [64,66]	**Desert** [60,64,67,68]; **Tropical** [66]
*Thielavia intermedia* Stchigel & Guarro	KJ117 KP941087 KP941101	35	20–40	4.9	95	**Saprophyte**: soil [68]	**Tropical** [68]
*Thielavia microspora* Mouch.	KJ123 KP941086 KP941100	30–40	15–50	11.8	52	**Saprophyte**: soil [68,69]	**Desert** [68,69]
*Thielavia subthermophila* Mouch.	KJ124 KP941085 KP941099	35	15–50	3.4	40	**Saprophyte**: soil [64]; **Endophyte** [64,68,70]	**Desert** [62,64,69]; **Temperate & tropical** [64,68,71]
**Hypocreales, Nectriaceae**							
*Fusarium chlamydosporum* Wollenw. & Reinking	KJ109 KP941094 KP941098	40	15–50	3.7	65	**Saprophyte:** soil [38,40,41,72,73]; **Endophyte** [74,75]; **Plant pathogen** [76]	**Desert** [40,41,56,72,77–79]**; Temperate & tropical** [75,76]
*Fusarium thapsinum* Klittich, J. F. Leslie, P. E. Nelson & Marasas	KJ116 KP941083 KP941097	25	10–35	5.0	64	**Endophyte** [80,81]; **Plant pathogen** [43,56]	**Desert** [56,80]; **Temperate** [43]
*Fusarium solani* (Mart.) Appel and Wollenw. emend. W.C. Snyder & H.N. Hansen	KJ115 KP941082 KP941096	25–30	15–35	5.0	59	**Saprophyte:** soil [73,77]; plant litter[45,67] **Endophyte** [82]; **Plant pathogen** [56,67,76]	**Desert** [34,41,47,53,56,78,79,83]; **Cosmopolitan** [67,77]

Thermal growth characteristics and previously known nutritional mode and habitat of fungi genotyped from standing *S*. *sabulicola* litter collected at Nara Valley and Station Dune in the Namib Sand Sea where fog is a frequent occurrence. KJCC Strain no. = Grinnell College Fungal Culture Collection housed at the Noyce Science Center, Grinnell, Iowa, USA; Genbank ITS Accession no. = internal transcribed region of the nrRNA amplified with primers ITS1/ITS4; Genbank SSU Accession no. = portion of the 18S nrRNA amplified with primers NS5/NS6; OGT = Optimal Growth Temperature.

**Fig 7:**
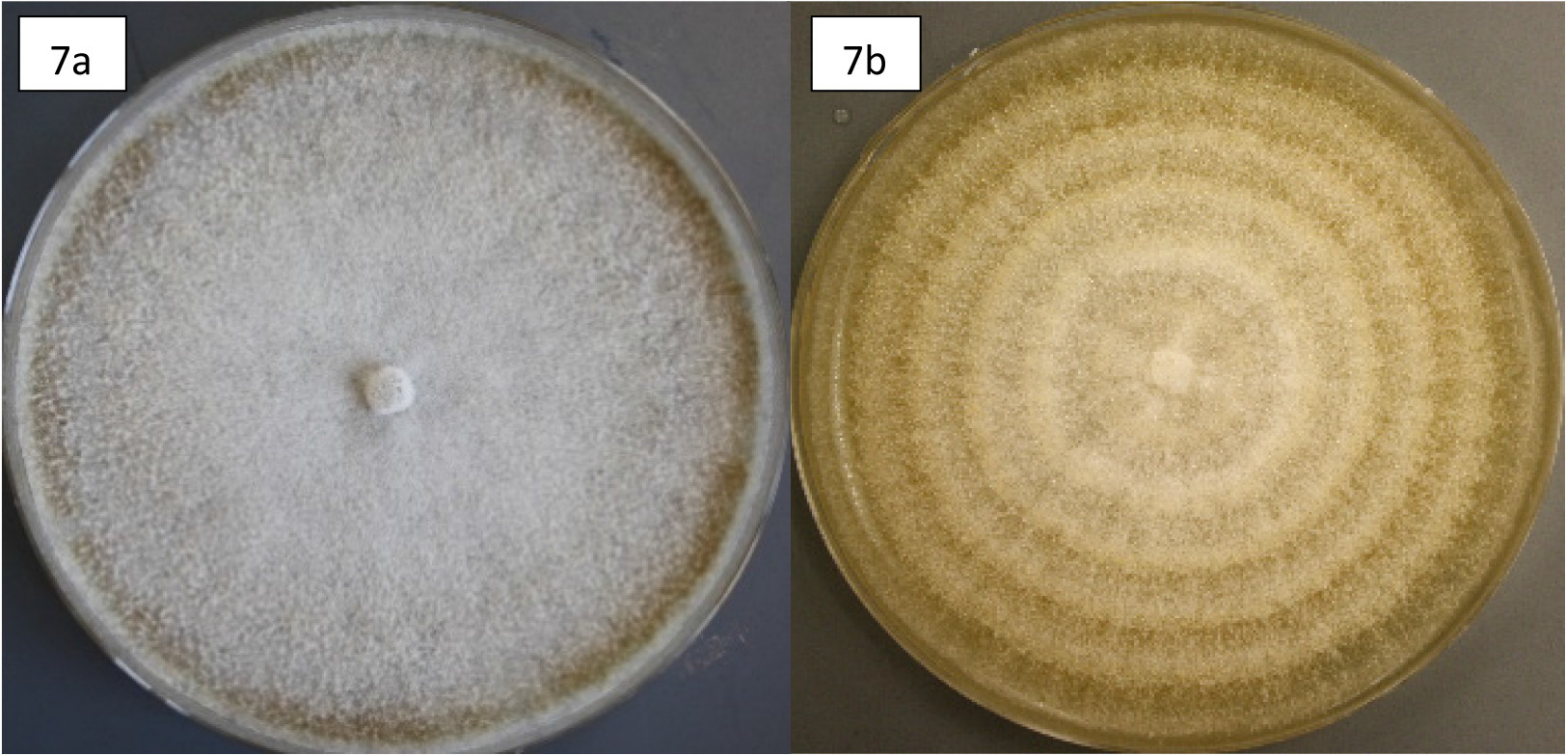
Diel thermal cycles induce fungal growth rings in culture. *Chaetomium strumarium* grown in the dark at optimal growth temperature (40⁰C) (7a); and with Namib diel sand-surface thermal cycle (15–50⁰C) (7b): 1 hour increments of increasing temperature from 20–45°C, 50°C for 5 hours, 1-hour increments of decreasing temperature from 45–25°C, and 20°C for 6 hours.

Optimal growth temperatures (OGT) for all 14 species ranged between 25–40°C (Table 2). Only 22% of the isolates had thermal optima at 40°C. All others had optima of 25–35°C. Two thirds of the isolates had similar optimal growth rates ranging from 3.4–5.2 mm d^-1^ (mean = 4.4 mm d^-1^). Three species (*Aspergillus niger*, *A*. *terreus*, *Aureobasidium pullulans*) grew significantly slower (1.8–2.2 mm d^-1^; T = 9.29, p<0.001) and three additional species (*C*. *atrobrunneum*, *C*. *strumarium*, and one isolate of *Thielavia microspora)* were significantly faster (11.8–12.1 mm d^-1^; T = 29.41, p<0.001). None of the fungi were thermophilic, defined as growing at temperatures above 50°C. Rather, 39% of the isolates were thermotolerant, with thermal ranges extending up to, but not exceeding, 50°C. The vast majority of the isolates (50%) are best defined as mesophilic with thermal ranges extending to 40°C, although two isolates were intermediate and able to tolerate growth at 45°C. Of note, 61% were able to grow at temperatures as low as 15°C, and 33% were able to grow at 10°C, and the low thermal limit for *A*. *pullulans* was below 5°C. In addition, all isolates continued growth without visible signs of cessation or aging (i.e. sectoring) for more than seven diel thermal cycles mimicking that of the sand-surface temperature experienced in the Namib (15–50°C). In all cases, growth rates were reduced from rates at optimum temperatures. The largest reductions were 95% for *Thielavia intermedia*, which also had the narrowest thermal range (20–40°C); and 88% for *Aureobasidium pullulans* which had a low thermal optima (25°C) and the lowest low limit of the thermal range. Growth reductions of the other isolates ranged from 33–65% (mean = 52%). All cultures developed growth rings, indicating changes in growth rate over the diel cycle (Fig 7A and 7B), and produced reddish-yellow to black pigments when grown above their thermal optima, at 35°C or above.

### 5. Substrate quality analysis

**Fig 8:**
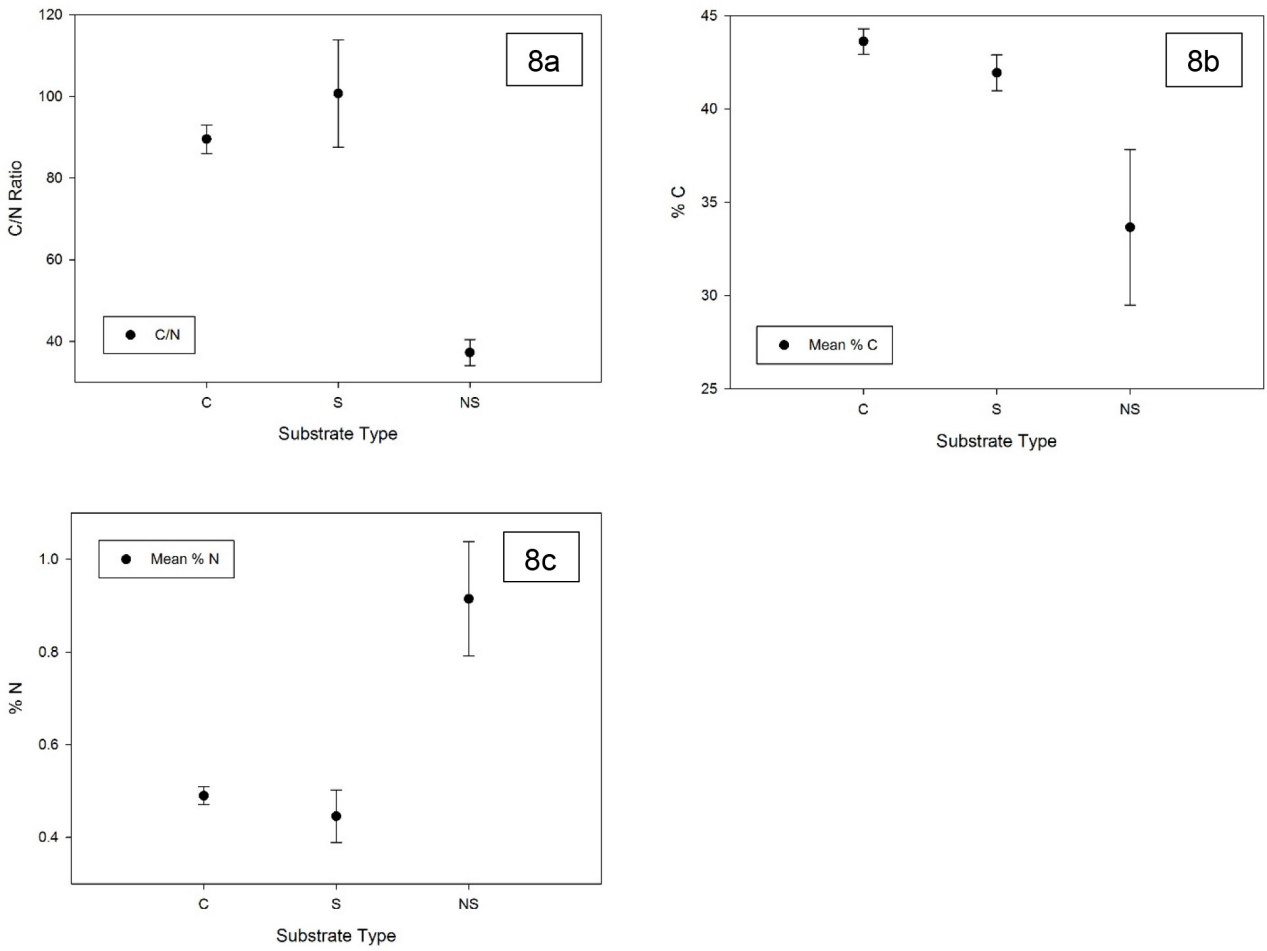
Fungal colonization enriches nitrogen content of colonized surface litter (NS) relative to that of uncolonized golden-yellow litter (C) and litter remaining after grey surface is stripped by termites (S). C/N ratios (8a) and %C (8b) of colonized litter were significantly lower than that of uncolonized and stripped litter. %N of colonized litter was significantly higher than that of uncolonized and stripped litter (n = 5; error bars = 1 SE; p<0.001).

The C/N ratios of the outer cortex of litter not yet colonized by fungi, and the litter core remaining in the casts that had been stripped, were not significantly different (p>0.05), averaging 89.6 and 100.7, respectively (Fig 8A). In contrast, the C/N ratio of the outer cortex of grey litter colonized by fungi was significantly lower, averaging 37.3. This was due to a decrease in percent C from 42% to 33.7% (p<0.001) in the grey colonized litter (Fig 8B), and a near doubling in the percent N from 0.47% to 0.92% (p<0.001) (Fig 8C). Thus, on a mass-specific basis, fungal colonization of substrates resulted in a significant N-enrichment of the outer layer consumed by termites in the casts.

## Discussion

### Fungal responses to different moisture sources

Our meteorological analysis shows that small precipitating fog and rainfall events of 1–2 mm that could wet the substrates in the same manner as our wetting experiments occur on average every 6–7 days, making these small moisture inputs a regular source of usable moisture for these surface fungi. While precipitation events of this magnitude are almost an order of magnitude too small to trigger below-ground decomposition (>9 mm required [4]), we found that they are sufficient to induce microbial metabolic activity on standing litter, supporting our initial hypothesis [H_1_]. This rapid response of free-living fungi to small moisture inputs has not been previously documented from the Namib but was predicted by Sterflinger et al. (2012) [84] regarding black extremophile hypolithic fungi in hot deserts, based on observations of lichens that respond “instantaneously” to small amounts of moisture [85].

Dirks et al. (2010) [20] conducted a decomposition experiment in the Mediterranean shrublands in which they observed 15–50% of annual litter loss during a moisture-free period (no rain or fog), which they attributed to microbial activity in response to night time periods of high humidity. Kuehn et al. (1998, 1999) [86,87] reported diel periodicity in respiratory activity of decomposing standing litter in wetlands, with the highest rates at night and early morning when standing litter had been wetted by high humidity and dew. The microbial response to wetting was rapid, with CO_2_ flux increasing to over 1 μg CO_2_-C g litter^-1^ min^-1^ within 5 minutes of wetting, similar to the response of standing litter in the NSS. Much is yet to be learned about the interactions of microbes with non-rainfall sources of moisture in the Namib. Based on this initial experiment we conclude that fungi on thicker standing stem litter require free water, which can occur with precipitating fog or dew, for rapid activation of metabolic activity. In contrast, microbes colonizing finer litter such as leaves and inflorescences can make use of <6 hour periods of >95 humidity that occur at night in the NSS on average every 3–5 days, and can likely use periods of >95% RH as brief as two hours, which occur even more frequently. While such patterns have previously been reported from standing litter in wetlands [86,87], this is the first report of such activity from a hyper-arid environment.

We thus conclude that free-living microbes, especially fungi, are an integral part of the standing litter decomposer community in the NSS. Moisture inputs, including rainfall, fog and high humidity, that can stimulate microbial metabolic activity are much more frequent than the highly erratic rain events needed to trigger primary production. Dew is likely an additional form of moisture that could result in free water on litter in the NSS [88]. Henschel & Seely (2008) [8] estimated that as much as 30% of the total free water that moistens NSS surfaces and sand may be in the form of dew that is not measurable with standard rainfall and fog collectors, and urged further study of this moisture phenomenon. If the initial measurements of dew are found to accurately reflect the importance of this moisture source in the Namib, we hypothesize that dew also triggers microbial decomposition in the eastern half of the NSS. This region rarely receives fog, mean annual rainfall is low (50–100 mm), and yet we have observed dry-season greying of standing grass litter.

### Fungal function, taxa and traits

The two-fold reduction in the C/N ratio (~95 to 37) indicates that the fungal community has changed the composition of the standing litter by catabolic processes that break down the plant cell wall polymers. Fungal hyphae have a C/N ratio of ~10 [89] and thus contribute to the higher relative N content of this litter layer, while also respiring CO_2_, thus lowering the relative amount of substrate carbon on a mass specific basis. Fungicide exposure suggests that fungi dominate this rapid response to wetting, accounting for up to ~80% of the observed carbon flux. Precipitating fog and small rainfall events which deliver only 1–2 mm of free water can thus regularly activate this litter microbial community. The prokaryotic component of this community has not been examined but this initial study suggests that it makes a substantially smaller contribution to the rapid respiratory response we observed when the substrates were moistened. Fungi are known to tolerate more desiccation stress than bacteria [84,90], which likely accounts for the apparent functional dominance of fungi in this microbial community.

We identified fourteen predominantly mesophilic fungal taxa from the grey grass litter. All of the genera and many of the fungal species isolated are known to be efficient degraders of cellulose, hemicellulose, pectins and tannins (see references associated with Table 2) and some possess laccases and peroxidases which are involved in lignin metabolism [91]. All grow well at the cooler temperatures associated with fog events: 10–25°C. While none of the fungi can grow above 50°C, most grew with “growth-ring” formations (Fig 7A and 7B) in a diel thermal cycle ranging from 15–50°C, albeit at rates averaging 50% less than their optimal growth rates. The diel cycle mimics temperatures on the sand surface [6], and we thus propose that the growth ring formation we observed is evidence that fungal hyphae colonizing standing litter undergo a daily period of estivation during the five hours (or more) of critically high temperature (up to 50°C) and extremely low moisture availability, followed by hyphal growth when suitable moisture and temperature conditions return.

Working with oligotrophic hypolithic and endolithic fungi, Steinberger et al. (2012) [84] proposed that rapid reactivation of metabolism might be one of the most important features that allow microcolonial fungi to thrive and function in arid environments. This is likely true of the Namib “fog fungi” too, even though they are not oligotrophic, having ready access to carbon and nutrients from the plant litter they occupy. In addition to rapid metabolic reactivation in response to minimal moisture inputs, we propose that equally important is the ability of these primarily mesophilic fungi to aestivate during daily periods of maximal stress. In the Namib diel cycle, metabolically-active fungal hyphae decomposing exposed grass surfaces are exposed simultaneously to heat, UV irradiation and desiccation stressors during the morning hours after the sun rises and fog dissipates. All three of these stressors are known to increase the formation of injurious reactive oxygen species that require fungal protective mechanisms [90]. The Namib fungal isolates produced reddish-yellow to black pigments when grown above their thermal optima, at 35°C or above. Melanins are very dark, and often black, pigments found in the fungal cell wall which are known to provide protection against UV radiation and desiccation stress [49,50,92,93]. Carotenoid pigments that likely account for the yellow-red to brown pigments of some of our thermally-stressed cultures are also thought to be a fungal stress tolerance response, serving as anti-oxidants [93] and UV filters, and can also stabilize membranes [94]. Furthermore, common fungal metabolites such as trehalose and mannitol are up-regulated during stress [90,95]. Trehalose stabilizes membranes and enzymes, thus playing an important role in anhydrobiotic responses of many organisms as they respond to thermal and desiccation stress by halting metabolism [96]. Mannitol and other small polyols that increase in cells in response to heat stress serve as intra-cellular osmolytes [93] and may thus be especially important in hyphal cells during the aestivation period.

### A reappraisal of fungi in drylands: implications for carbon cycling and nutrient turnover

The important role that fungi can play in arid ecosystems was recently captured in the Threshold-Delay Nutrient Dynamics (TDND) theoretical model proposed by Collins et al. (2008) [19]. The authors propose that a fungal loop (comprised of soil decomposers, mycorrhizal and endophytic fungi, as well as fungi associated with biotic soil crusts), mediates plant response to pulsed precipitation by tightly linking C and N cycling, NPP and decomposition. Underlying assumptions of the model that differentiate it from those in more mesic systems are that physicochemical processes limit the incorporation of above-ground litter into below-ground soil organic matter accumulation and that the pulsed nature of moisture inputs and resultant desiccation stress limits the activity of prokaryotic microbes. While effectively highlighting the importance of soil-mediated fungal processes in arid ecosystems, our results suggest some important changes are needed for effectively modelling C and N dynamics in the NSS, and likely in other drylands as well. Firstly, the TDND model assumes that the only sources of metabolically-useful moisture for fungi are rainfall events sufficient to initiate primary production and moisten desert soils. Secondly, the authors assume that all arid land fungi are in the soil or associated with living plants. In contrast, we found that the fungal community decomposing surface plant litter in the Namib has a much lower moisture threshold for metabolic activity (1–2 mm) than previously understood. In addition, the fungi that comprise this community and occur in other drylands have the necessary traits to tolerate thermal, UV and desiccation stress in surface desert environments. Furthermore, Dirks et al. (2010) [20] and Gliksman et al. (2014) [97] have proposed that microbial decomposition in drylands occurs above-ground in association with high humidity in the absence of precipitation, both independently and in conjunction with UV-induced breakdown of lignocellulose. Other authors [98,99] have also suggested that these biotic and abiotic drivers work synergistically, but assumed that large infrequent precipitation events were required to activate the microbial activity.

A further assumption of the model is a tight loop of nutrient turnover amongst fungi and plants that excludes detritivores, which are also important agents of soil organic matter turnover in detritus-driven ecosystems [3]. Termites in particular, play an essential role in consuming litter in drylands, as well as moving it below-ground [7,100,101]. Our C/N analysis and field observations supported our second hypothesis [H_2_] that fungi enrich the outer-layer of standing stem litter and then, along with the litter, are consumed by the termite *Psammotermes allocerus*, thus disrupting the tight fungus-plant loop of N cycling proposed in the TDND model. Given the importance of termites in this desert and others, this detritivore component may be a substantial connection linked to the fungal loop. In addition to *P*. *allocerus* consuming grey *S*. *sabulicola* stems, we have also observed a second termite species (*Hodotermes mossambicus*) that cuts annual grass in the interdune plain using grey stems; as well as tenebrionid beetle larvae consuming fine windblown grey leaf and inflorescence detritus in buried accumulations associated with grass hummocks and slipfaces (Fig 1A). Once buried, this litter becomes a critical resource for larvae of the rich beetle communities in the Namib [3].

The importance of fungal-mediated litter N enrichment to beetle nutrition is unknown but given that all invertebrates use chitinases to molt, they possess the genetic machinery required to digest fungal hyphae [102]. We thus hypothesize that, like termites, all invertebrate detritivores in the Namib are feeding to some degree on fungal-enriched litter. Microbial colonization of litter is known to strongly influence foraging patterns of detritivores across a range of other ecosystems and climates. Fungal priming of leaf surfaces that are subsequently grazed by aquatic invertebrates is well known from stream habitats [103,104], where fungi increase the protein content, concentrate N relative to C and transform plant polymers into digestible forms, in addition to being eaten directly by the invertebrates. A similarly important role of fungi in terrestrial invertebrate nutrition is assumed [102] but only well-studied in certain taxa. For termites feeding on lignocellulosic substrates (e.g. wood), fungal hyphae are an important source of nutrients [105,106]. Chitinase activity and feeding behaviour have not been studied in *P*. *allocerus*, but other members of the Rhinotermiditae have gut chitinases [107].

Because the termite *P*. *allocerus* accounts for more than half of the litter consumed in the NSS [7], our study suggests that the influence of microbial conditioning of litter is a previously unrecognized yet important characteristic of N-cycling and energy flow in the Namib that deserves further attention. Current efforts are underway at Gobabeb to more accurately measure complex moisture dynamics, including rainfall, fog, high humidity and dew, in light of forecast climatic changes associated both with changing Atlantic Ocean surface temperatures and regional climates across southern Africa [108,109]. Moisture source dynamics in the Namib are complex, with contributions from both the Atlantic (primarily advective fog) and the Indian Ocean (rainfall). The origins of moisture associated with radiation fog and dew are unknown, but both oceans contribute water and thus climate change may exert profound influences on the region, especially given the functional significance of small amounts of non-rainfall moisture to microbes. Given the importance of these different moisture sources to both above and below-ground decomposition processes, refinement of energy flow and nutrient-cycling models is needed. Arid and semiarid lands account for 40% of global terrestrial landscapes [110], yet efforts to model nutrient dynamics consistently underestimate decomposition rates in arid ecosystems [99]. We documented that fungi are capable of decomposing litter in response to minimal free water additions or 2-hours of high humidity. Given the importance of litter as a source of energy and nutrients in deserts [2], we propose that fungal decomposition of surface litter in association with minimal moisture events accounts for a previously unknown, yet important component of desert decomposition processes and associated food webs.
